# ASO Author Reflections: Outcomes Support Aggressive Surgery for Early-Onset Pancreatic Cancer

**DOI:** 10.1245/s10434-022-12948-5

**Published:** 2022-12-26

**Authors:** Carl-Stephan Leonhardt, Benedict Kinny-Köster, Jin He, Oliver Strobel

**Affiliations:** 1 Department of General, Visceral and Transplantation Surgery, Heidelberg University Hospital, Heidelberg, Germany; 2 Present Address: Department of General Surgery, Division of Visceral Surgery, Medical University of Vienna, Vienna, Austria; 3 Department of Surgery, Johns Hopkins University School of Medicine, Baltimore, MD USA; 4 Department of Surgery, New York University Grossman School of Medicine, NYU Langone Health, New York, NY USA

## Past

Similar to other tumor entities including colorectal cancer, the incidence of early-onset pancreatic cancer (EOPC) affecting individuals ≤ 45 years of age is on the rise.^1^ While causes remain mostly unclear, clinicians are more and more frequently confronted with this young patient population in daily practice. Little is known about clinical outcomes in patients with EOPC, and age at diagnosis is not considered in current treatment guidelines. In our manuscript titled “Resected Early-Onset Pancreatic Cancer: Practices and Outcomes in an International Dual-Center Study” we investigate clinicopathologic features as well as outcomes of patients with EOPC undergoing resection.^2^

## Present

In a dual-center study between Heidelberg University Hospital and Johns Hopkins Hospital, we present the largest series of patients with EOPC after surgery. We demonstrate that oncologic outcomes of patients with EOPC are excellent in stage I tumors, with a median overall survival of 70 months and reaching a median survival of 29.5 months even in locally advanced disease (T4N0/1). Classical prognostic factors of resected pancreatic cancer, including AJCC tumor stage, pN stage, and margin status, were confirmed. After upfront surgery, 85% of patients with EOPC received adjuvant chemotherapy, a noticeably higher rate than reported after average-onset pancreatic cancer. However, in both centers a trend toward neoadjuvant treatment was observed, although preoperative staging of tumors remained constant.

## Future

A number of questions remain and need to be addressed in future research efforts. First, the contributions of hereditary pancreatic cancer and of molecular aberrations in the etiology and biology of EOPC need to be investigated. On a larger scale, the rising incidence of pancreatic cancer affecting individuals ≤ 45 years of age demands further attention from epidemiological scientists to investigate the associations of macroenvironmental risk factors in EOPC carcinogenesis. A comprehensive assessment of molecular characteristics may identify potentially actionable EOPC-specific vulnerabilities for which novel personalized systemic therapies are emerging.^3^ Dedicated prospective studies comparing oncologic outcomes as well as patient-reported outcomes between EOPC and average-onset pancreatic cancer are necessary to assess if a more aggressive treatment including aggressive systemic therapy and extended resections should be recommended in treatment guidelines for patients with EOPC.
